# Job requirement level, work demands, and health: a prospective study among older workers

**DOI:** 10.1007/s00420-019-01451-2

**Published:** 2019-06-12

**Authors:** Karolin Hiesinger, Silke Tophoven

**Affiliations:** 1 Institute for Employment Research, Regensburger Str. 104, 90478 Nuremberg, Germany; 2 Present Address: City of Krefeld, 47792 Krefeld, Germany

**Keywords:** Psychosocial work demands, Physical work demands, Physical health, Mental health, Mediation, Older workers

## Abstract

**Purpose:**

Persons in lower occupational positions experience higher rates of morbidity compared to workers in higher advantaged positions. Working conditions may explain this occupational health gradient. Most studies consider either psychosocial or physical work demands at one point in time. In our study, we examine both physical and psychosocial work demands and their association with health status differentiated by job requirement level. We further distinguish between constant and changing work demands.

**Methods:**

Using data from the first two waves of the German cohort study on work, age and health, we analyse a sample of 3644 older workers born in 1959 and 1965. We test direct and mediating effects of high physical and psychosocial work demands on functional physical and mental health. For this, we estimate a prospective path model using multiple linear regression models.

**Results:**

Our results show that (1) constant high physical and psychosocial work demands affect physical and mental health negatively and (2) high physical workload partly mediates the relationship between job requirement level and physical health. Moreover, at least for men, a reduction of physical and psychosocial workload improves mental health status.

**Conclusions:**

Research and prevention measures currently focus particularly on psychosocial work demands. Our study shows that high physical workload is still present among older workers. Its negative health effect refers to occupational safety and health measures that take into account both the physical and psychosocial work environment as well as workers’ occupational positions.

## Introduction

A social gradient in health is well documented in empirical research: groups with a lower occupational status experience higher rates of morbidity compared to higher advantaged groups of workers (Mackenbach et al. 2008; van der Molen et al. 2018). In addition, a social gradient is observable not only for health, but also for job quality and work exposures (Dragano and Wahrendorf 2016). Focusing on work environment and working conditions, the mediation hypothesis serves as one explanation for this gradient: workers experience different levels of strenuous work demands depending on their occupational position (Clougherty et al. 2010; Hämmig and Bauer 2013; Hoven and Siegrist 2013). Strenuous work demands, in turn, affect physical and mental health negatively (Kivimäki et al. 2006; Nyberg et al. 2014; Rugulies et al. 2013).

Empirical studies identify a mediating effect of physical work demands for the relationship between occupational position and physical health (Kaikkonen et al. 2009; Lahelma et al. 2006; Niedhammer et al. 2008): workers with a lower occupational position experience health-adverse working conditions more often, as their jobs are rather characterized by unhealthy risk factors (Hämmig and Bauer 2013). They have, for example, a higher risk for injury and exposure to chemical and biological hazards (Mehlum et al. 2008; Montano 2014).

The picture is less clear with regard to psychosocial work demands, as some harmful risk factors such as high time pressure, frequent interruptions, or working overtime are more likely in higher occupational positions (Hoven and Siegrist 2013; Kaikkonen et al. 2009; Lahelma et al. 2006). Other strains such as monotonous work, inflexible working hours, and low job autonomy remain to be more prevalent in lower occupational positions (Hämmig and Bauer 2013; Niedhammer et al. 2008). Currently, research as well as preventive measures focus mainly on psychosocial work demands (Eurofound and EU-OSHA 2014). However, physical work demands are still prevalent and may be even more health damaging than psychosocial factors, particularly for certain occupational groups (Knudsen et al. 2018; Monden 2005; Parent-Thirion et al. 2012). The present paper, therefore, aims to simultaneously analyse the direct and indirect effects of both physical and psychosocial work demands on health among older workers. Using the first two waves of the lidA study, we perform a mediation analysis in a prospective design on functional physical and mental health status. Our independent variable of interest is occupational position. We include high physical and psychosocial work demands as possible mediators for this association and differentiate between acute and constant workload as well as a reduction in workload.

So far, many studies are cross-sectional and focus on work demands at one point in time. This focus neglects the dynamics in the prevalence of strenuous working conditions and their impact on health, because the previous events and conditions may also affect current health status (Blane 2006; Wahrendorf and Chandola 2016). The few existing empirical studies indicate that both high work demands at one point as well as high workload over a longer period of time influence health negatively, while a reduction of workload results in improved health (de Lange et al. 2002; Godin et al. 2005). Again, the dynamics in the prevalence of workload differ with occupational position and, therefore, explain occupational health inequalities: workers in lower positions do not only experience more harmful working conditions at one point in time, but also an increase in these working conditions over the life span (Blane 2006; Monden 2005; Wahrendorf et al. 2013). In addition, the differences in working conditions between occupational groups increase over the life span. The occupational gradient in health is, therefore, more pronounced for older workers (Chandola et al. 2007).

In the face of demographic change, a longer and better integration of particularly older workers among all occupational positions into the labour market is necessary. Since healthier workers stay in the workforce longer, creating a health-promoting work environment and reducing health inequalities are important current challenges to enable long working lives (Stattin 2005). Our study, therefore, aims to contribute to a better understanding of the underlying mechanisms of health inequalities in older working age.

## Methods and data

### Data

We use data from the first two waves of the German lidA study, a cohort study on work, age, and health. The lidA sample comprises a representative sample of German employees born in 1959 and 1965 subject to social security contributions. In 2011, 6585 persons answered to questions concerning issues of work and health in a personal interview. Three years later, in 2014, 4244 of these persons were interviewed again (Hasselhorn et al. 2014). For our analyses, we use information from the 3644 persons who answered to the relevant questions in both waves.

### Variables

#### Physical and mental health

The lidA study measures self-rated functional mental and physical health with a 12-item validated health questionnaire (SF-12). We standardize the values by the recommended norm-based scoring. As a result, the values are comparable to a representative German population in 2004 (Andersen et al. 2007). The values range from zero to 100, with 50 describing the average of the population in 2004; hence, higher (lower) values correspond to better (worse) health than the average.

#### Job requirement level

We use job requirement level as an indicator for occupational position. Within the data, occupations are classified according to the German classification code of 2010. This classification scheme has two dimensions: the horizontal dimension classifies occupations according to their similarity of required skills, abilities, and knowledge. The vertical dimension depicts the various degrees of complexity of similar occupations. We make use of the latter. The vertical dimension distinguishes four requirement levels mainly defined by formal vocational qualification: unskilled or semiskilled activities (no vocational qualification or regular 1-year vocational training), specialist activities (at least 2 years of vocational training), complex specialist activities (qualification as master craftsman or technician, graduation from a professional academy or university bachelor’s degree), and highly complex activities (completed university studies of at least 4 years) (Paulus and Matthes 2013). Albeit guided by formal vocational qualifications, the job requirement level required for practicing an occupation can also be acquired through work experience and training on the job.^1^

#### High psychosocial workload

Psychosocial workload is captured in reference to the established “effort–reward imbalance (ERI) model” using a validated questionnaire with 22 single items (5 on efforts, 11 on rewards, and 6 on overcommitment) (Siegrist 1996, 2016). This model not only focuses on demanding psychosocial work requirements, including being compelled to work overtime and under high pressure, but also considers rewards such as financial, status-related, and socioemotional benefits. Therefore, the ratio between “costs” in terms of spent efforts and “gains” in terms of received rewards is crucial: an imbalance due to high efforts and low rewards leads to adverse health effects. We form an index describing the ratio of efforts and rewards and weighted by the respective number of efforts and rewards. Following previous studies, we use the relative level of the index and classify persons, whose index values are in the highest quartile of the distribution as those who experience high psychosocial workload (Godin et al. 2005; Siegrist et al. 2004).

#### High physical workload

The degree of physical work demands is measured on the basis of three items describing working conditions with regard to ergonomic strains (Kroll 2011; Tophoven et al. 2016).^2^ The respondents were asked on a five-stage scale how often (never, up to one quarter of the time, up to half of the time, up to three quarters of the time, and more than three quarters of the time) they are exposed to the following demands.

- Working sitting down.
- Working in a bending, squatting or kneeling position, and lying down or above your head.
- Lifting and/or carrying heavy loads.

For the item “working sitting down”, we inverted the scale.^3^ We consider those persons experiencing physical demands more than one-quarter of the time on average to be strained by high physical workload (Kroll 2011).

### Statistical analyses

Our analysis follows a prospective study design (Godin et al. 2005). Our dependent variable is functional health status at wave two of the survey (t2). We run separate analyses for functional physical and mental health. To identify changes in psychosocial as well as physical workload, we include both timepoints and distinguish four categories for each type of workload: (1) no high work demands at t1 and t2; (2) high work demands at t1 but not at t2; (3) high work demands at t2 but not at t1; and (4) high work demands at t1 and t2. This approach allows us to analyse how acute and constant workload as well as a reduction of workload affect prospective functional health status. We further control for year of birth, working time (full time versus part time), and functional physical health status in the first wave.

**Fig. 1:**
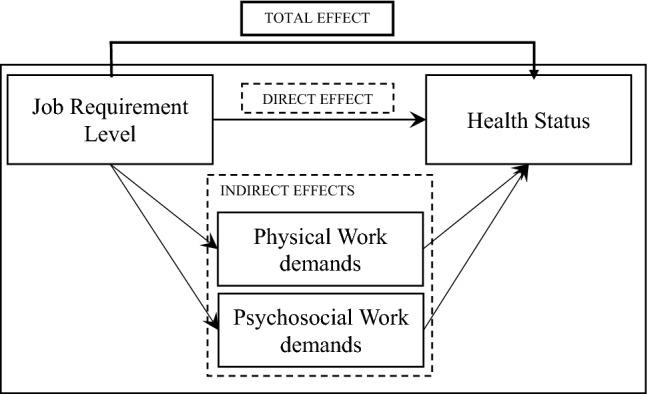
Illustration of the mediation analysis

Figure 1 describes our approach of the mediation analysis following Baron and Kenny (1986): first, we regress functional health status (dependent variable) on job requirement level (independent variable) in a linear regression to obtain the total effect of occupational position on health. Second, we analyse the effect of job requirement level (independent variable) on changes in psychosocial and physical work demands (mediator variables). We include the four categories of the mediator variables as dummy variables (in reference to the category “no high level in both waves”) and estimate linear probability models. The resulting coefficient describes the probability of being in the observed category compared to persons of the reference category (i.e., specialists). Finally, we regress functional health status on job requirement level and the mediators (psychosocial and physical work demands). This approach allows us to decompose the total effect into (a) the direct effect of job requirement level on health status and (b) the indirect effects of this association mediated by high physical and psychosocial demands. To identify gender-specific differences, we run the regressions stratified for gender. Using Stata 14.2 enables to perform the described regressions simultaneously (Acock 2013). Model fit is evaluated in accordance with the standardized root mean squared residual (SRMR) and the determination coefficient (*R*^2^). The recommended SRMR value is less than 0.08 (Hu and Bentler 1999). All presented models have a satisfactory model fit.

## Results

### Descriptives

The study population comprises 1978 (54.3%) females and 1666 (45.7%) males. The lidA study focuses on older workers born in 1959 and 1965. The share of those born in 1965 is higher than the share of the older cohort (55.9%–44.1%). With regard to job requirement level, the majority works in specialist activities (55.4%), while only 248 (6.8%) persons work in unskilled or semiskilled activities. A total of 669 (18.4%) respondents work as complex specialists, and 708 (19.4%) in jobs categorized as highly complex activities.

**Fig. 2:**
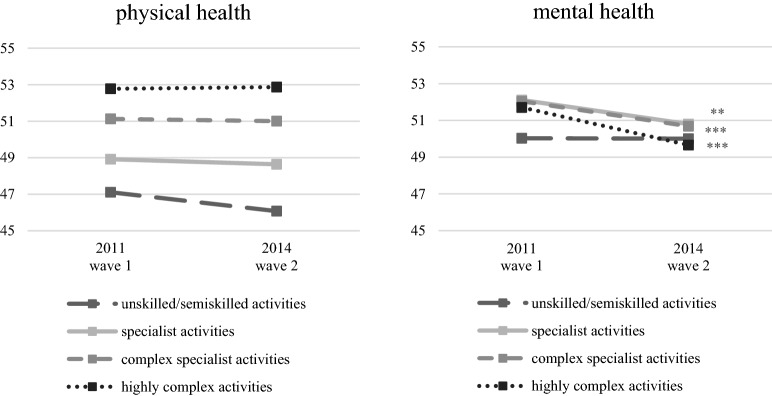
Physical and mental health dependent on job requirement level. Means of scores for physical and mental health measured with the SF-12; SF-12-values range between 0 and 100, whereby 50 is the average of a normed population; *n* = 3644; *t* test of differences in health score between the two waves: *p < 0.05, **p < 0.01, ***p < 0.001. Source: lidA waves 1 and 2, own calculations

Within the sample, both functional physical and mental health status differ with regard to job requirement level (Fig. 2). In terms of physical health, we find a clear occupational health gradient in both waves, whereas the pattern is ambiguous for mental health.

**Fig. 3:**
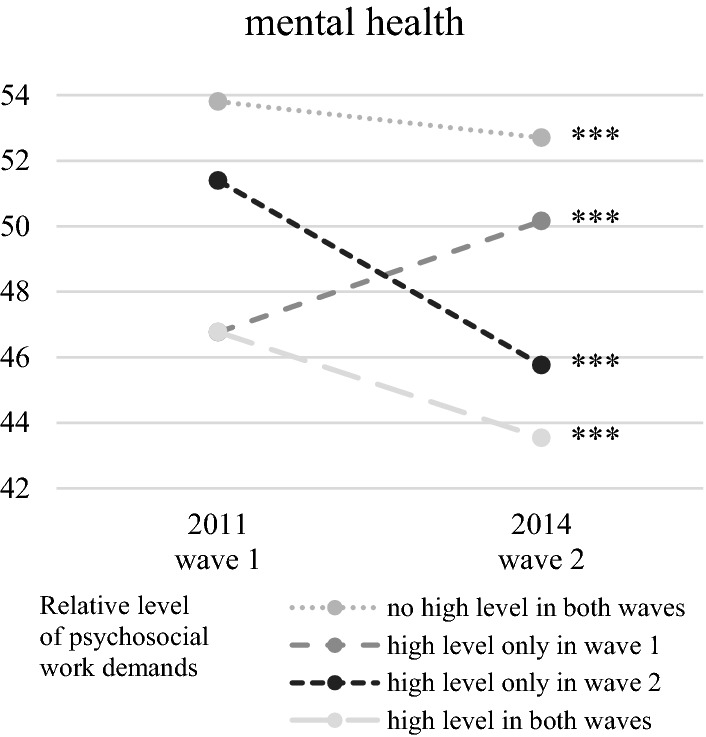
Mental health dependent on the level of psychosocial work demands. Means of scores for mental health measured with the SF-12; SF-12-values range between 0 and 100, whereby 50 is the average of a normed population; *n* = 3644; *t* test of differences in health score between the two waves: *p < 0.05, **p < 0.01, ***p < 0.001. Source: lidA waves 1 and 2, own calculations

**Fig. 4:**
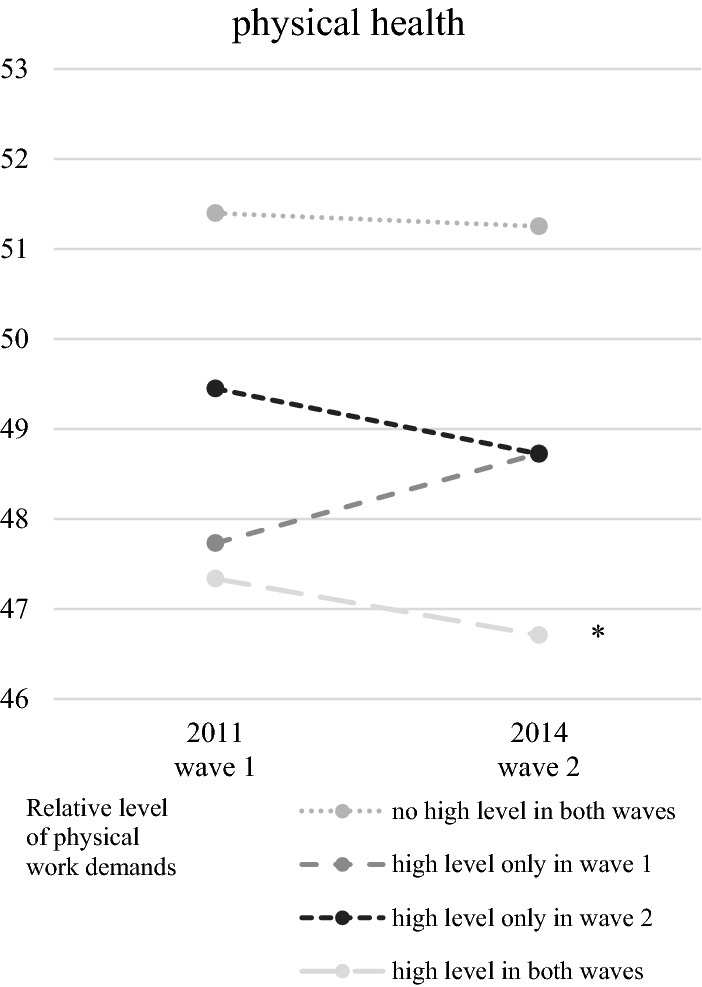
Physical health dependent on the level of physical work demands. Means of scores for physical health measured with the SF-12; SF-12 values range between 0 and 100, whereby 50 is the average of a norm population; *n* = 3644; *t* test of differences in health score between the two waves: *p < 0.05, **p < 0.01, ***p < 0.001. Source: lidA waves 1 and 2, own calculations

In terms of work demands, many workers do not experience high demands in the first or second wave (65.5% for psychosocial and 60.2% for physical demands). A total of 470 (12.9%) workers are strained constantly by high psychosocial work demands in both waves, while 951 (26.1%) workers experience high physical work demands in both waves. The share of persons reporting high work demands at one point in time is similar for both waves. About 11% of the study participants report high psychosocial work demands, and 7% report high physical work demands in wave 1 or wave 2. Figures 3 and 4 illustrate that the level of work demands is associated with physical and mental health.

### Multivariate analyses

#### Direct effects on prospective physical health status

In the multivariate analyses with physical health as dependent variable, the direct effects of job requirement level show the expected directions (Table 1): on the health scale ranging from 0 to 100, persons in unskilled or semiskilled activities on average have a 1.33 point worse physical health status in reference to specialists. The health status of workers in complex specialist positions is on average 0.96 points better in reference to specialists. The health status of workers with highly complex activities is 1.80 points better in reference to those holding specialist positions. These differences are statistically significant and controlled for work demands, high overcommitment, year of birth, working full time, and physical health score in wave 1.

**Table 1:** Multivariate analyses: direct effects on prospective physical health status. Source: lidA waves 1 and 2, own calculations

	Dependent variable: physical health score in wave 2		
	Whole sample	Men	Women
Direct effects			
Job requirement level^a^			
Unskilled or semiskilled activities	− 1.33*	− 1.95	− 1.02
Complex specialist activities	0.96**	1.14*	0.79
Highly complex activities	1.80***	1.79***	1.86***
Level of psychosocial work demands^b^			
High level only in wave 1	− 0.06	− 0.68	0.59
High level only in wave 2	− 1.82***	− 1.77*	− 1.76**
High level in both waves	− 1.24**	− 1.10*	− 1.26*
Level of physical work demands^b^			
High level only in wave 1	− 0.02	− 0.24	0.14
High level only in wave 2	− 0.97	− 1.25	− 0.75
High level in both waves	− 1.54***	− 1.23**	− 1.83***
High overcommitment	− 0.14	0.41	− 0.59
Year of birth: 1959	− 0.94***	− 1.14**	− 0.74*
Working full time	− 0.49	0.39	− 0.82*
Physical health score in wave 1	0.53***	0.54***	0.52***
Coefficient of determination (R^2^)	0.50	0.52	0.52
Standardized root mean squared residual (SRMSR)	0.04	0.04	0.04
*N*	3644	1666	1978

The analyses are based on multiple linear regression analyses. The table shows unstandardized regression coefficients. The total indirect effects are not shown in this table. The detailed table of results with direct and indirect effects can be found in the Appendix (Table 3)

*p < 0.05, **p < 0.01, ***p < 0.001

^a^Reference category: specialist activities

^b^Reference category: no high level in both waves

With regard to work demands, the results demonstrate that both acute and constant high psychosocial work demands affect physical health negatively. With regard to physical demands, only constant high physical demands have a significant negative effect on physical health.

#### Mediating effects

**Fig. 5:**
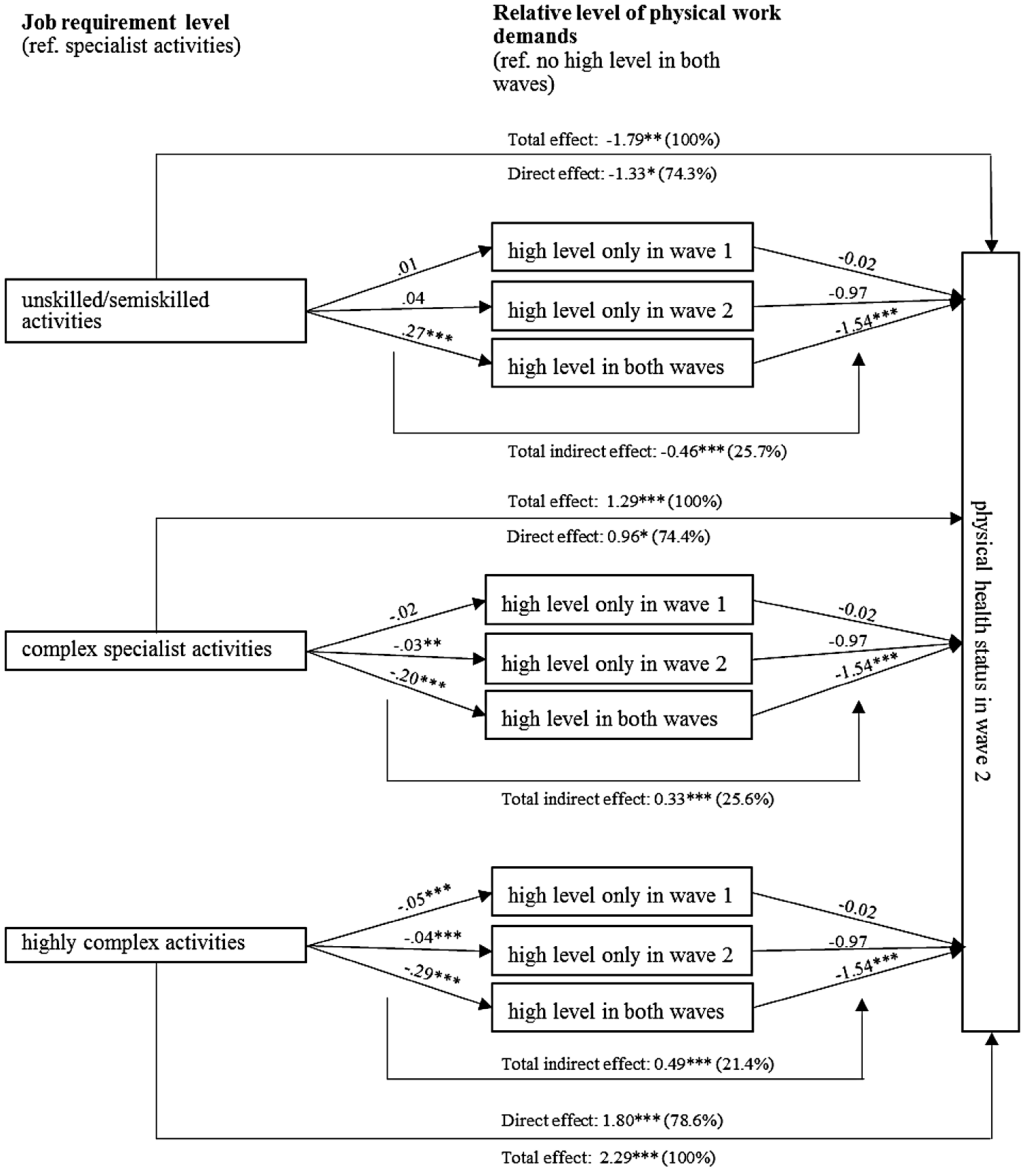
Physical work demands as a mediator of the association between job requirement level and prospective physical health status. *n* = 3644; * *p* < 0.05, ** *p* < 0.01, *** *p* < 0.001. The direct, total indirect effects, and the effects of high work demand on physical health represent the multivariate regression results, as reported in Tables 1 and 3. The total effect is the effect of a simple regression of physical health on job requirement level. The effects of job requirement level on work demands stem from linear probability models estimating the probability of being in one of the categories compared to the reference category. Reading example: compared to specialists, people in unskilled jobs are 27 percentage points more likely to be exposed to high physical workloads in both waves. Figure 6 in the appendix shows the odds ratios for the associations. Source: lidA waves 1 and 2, own calculations

High physical work demands partly mediate the association between job requirement level and physical health. Figure 5 shows the total, direct, and indirect effects of the association between job requirement level, high physical work demands, and physical health for the whole sample in detail: persons in unskilled or semiskilled activities have a higher probability of experiencing constant high physical workload compared to those in specialist jobs. As discussed previously, high physical work demands, in turn, influence physical health status negatively. In contrast, persons in complex specialist or highly complex activities are less likely to be affected by acute and constant high physical workload, which contributes to their better physical health status on average.

#### Direct effects on prospective mental health status

With regard to mental health, we identify a significant effect of requirement level only for workers in highly complex activities (Table 2). They have a significantly worse mental health status at the second wave compared to specialists. The overall differences between the categories of job requirement level are smaller compared to the model with physical health as dependent variable. As a result, we cannot identify a clear occupational gradient in mental health which makes a further mediation analysis unnecessary.

**Table 2:** Multivariate analyses: direct effects on prospective mental health status. Source: lidA waves 1 and 2, own calculations

	Dependent variable: mental health score in wave 2		
	Whole sample	Men	Women
Direct effects			
Job requirement level^a^			
Unskilled or semiskilled activities	− 0.38	− 0.63	− 0.06
Complex specialist activities	− 0.14	− 0.13	− 0.20
Highly complex activities	− 0.84*	− 0.59	− 1.15
level of psychosocial work demands^b^			
High level only in wave 1	0.66	1.37*	0.21
High level only in wave 2	− 5.68***	− 5.65***	− 5.48***
High level in both waves	− 5.76***	− 5.16***	− 6.00***
Level of physical work demands^a^			
High level only in wave 1	1.21	2.01**	0.48
High level only in wave 2	− 0.42	− 0.42	− 0.36
High level in both waves	0.06	0.57	− 0.53
High Overcommitment	− 1.74***	− 1.64**	− 1.65**
Year of birth: 1959	0.79*	1.21**	0.50
Working full time	1.44***	0.92	0.58
Mental health score in wave 1	0.38***	0.43***	0.34***
Coefficient of determination (*R*^2^)	0.26	0.31	0.21
*N*	3644	1666	1978

The analyses are based on multiple linear regressions analyses. The table shows unstandardized regression coefficients

*p < 0.05, **p < 0.01, ***p < 0.001

^a^Reference category: specialist activities

^b^Reference category: no high level in both waves

Constant and acute psychosocial work demands harm mental health significantly. These effects are much greater than those on physical health are. Moreover, for men, we can identify a significant positive effect stemming from the reduction of both psychosocial and physical workload on mental health.

## Discussion

Nowadays, both research and health interventions mainly focus on psychosocial workloads. Because of the changing world of work due to digitisation and automation, this spotlight seems justified: psychosocial workloads are assumed to gain importance, while physical workloads are losing importance (Arnold et al. 2017). Our study among older workers confirms that psychosocial workloads are present in all occupational positions and have a negative impact on both mental and physical functional health. In addition, we could also show that physical work demands are still prevalent and mediate the relationship between occupational position and physical health.

Given the high relevance of physical work demands for health and for explaining health inequalities, the one-sided focus on psychosocial workloads does not reflect the working reality of all older workers in Germany. This applies in particular to workers in lower occupational positions. Our findings identify those workers as a multiple disadvantaged risk group in the working population: first, they have an on average poorer health status than persons in higher positions. Second, their jobs are more often characterized by constant physically demanding strains, which additionally influence the health status negatively.

Measures to promote the health of older workers should, therefore, primarily target workers in lower occupational positions. Such measures may include, for example, the implementation of new digital technologies that make physically demanding work easier. In this process, however, it is important that particularly older workers do not feel detached. Qualification and training in the use of these new technologies are important instruments to maintain and promote the employability of older low-skilled workers in the future. Equipped with the appropriate skills, those workers may perceive the digital change not as a further disadvantage, but as their chance for a healthy and long working life.

Caring for healthy physical and psychosocial working conditions, therefore, contributes not only to a better health among workers, which enables them to stay longer in the workforce; it also helps to reduce health inequalities during work life and beyond. An improvement in working conditions pays off: at least among men, our results show that a reduction in physical and psychosocial workload significantly improves mental health.

Due to digitisation, the current world of work will continue to change. Occupations themselves and the distribution of occupational positions will become different (Weber 2017). The development of new technologies may further change the physical and psychosocial work environment in the future for all workers and in particular for those in lower occupational positions. As discussed above, new technologies may be an opportunity to improve the physical work environment of low-skilled workers. This improvement could lead to a decreasing occupational health gradient. Contrary, new technologies also create new risks for occupational safety and health (European Agency for Safety and Health 2017). An important task for future research is to investigate the development of physical and psychosocial workloads and their influence on health and health inequalities in the face of digitisation. In doing so, it is also important to consider further health-related outcomes in addition to the self-reported health status, for example, work-related diseases (van der Molen et al. 2018).

## Limitations to the study

The present study has some limitations that have to be mentioned. First, our sample includes only persons who took part in the lidA study at both measurement points in 2011 and 2014 and who were employed at both points in time. Hence, the participants are a selective sample of German workers born in 1959 and 1965. Persons in the observed age groups who are strained by high workloads may have already left the labour market and have not taken part in the survey. In Germany, the mean age of people who start drawing disability pension is 52 in Germany (Deutsche Rentenversicherung Bund 2017). As a result, a healthy worker effect may bias our results.

Second, as we only have two measurements points (2011 and 2014) available, we cannot identify any long-term effects of (changing) work demands. In addition, we cannot cover the previous history of working conditions and health status.

Third, in terms of effect size, the significant effects of job requirement level on physical health are not that large per se. However, when comparing the differences between the two groups at the margins—workers in unskilled or semiskilled activities and workers in highly complex activities—the health gradient is quite substantial. To check the robustness of our results, we performed two additional analyses. First, we estimated an alternative model using educational level instead of job requirement level as independent variable. The model including job requirement level has more explanatory power and captures the more relevant categories in terms of occupational position than the model including the education variable. Second, we estimated an expanded model including regions and occupational segments as further occupation-specific control variables and migration background and family status as further sociodemographic control variables. The results confirm our main results.

Finally, the risk of reversed causality cannot be fully excluded as work demands and outcomes are simultaneously measured at the second wave. It is, therefore, not possible to interpret the effects found in our study as true cause-and-effect relationships. Despite the mentioned limitations of our study, the used data of the lidA study enables a prospective analysis of functional health status including a rich set of relevant factors to test our hypothesis focussing on older workers.

## Conclusion

The present study shows that high psychosocial workload affects physical and particularly mental health among older workers negatively. In addition, over 25% of our study population report permanently high physical workload, which affects physical health negatively. The exposure to physical work demands is strongly dependent on the job requirement level: unskilled or semiskilled workers have a significantly higher risk to perceive high physical work demands. Occupational safety and health measures should, therefore, not only focus on psychosocial, but also on physical risk factors of the different occupational groups, in particular workers in lower occupational positions. A reduction of high physical workload contributes to both a healthy ageing workforce in general and to a reduction in social health inequalities. For future research, a long-term panel study—enabling work history analyses in terms of work demands and including persons in the labour market and those who have already left the labour force—would provide even deeper insights into how changes in work demands causally affect health and how these demands can explain health inequalities.

## Figures and Tables

**Fig. 6:**
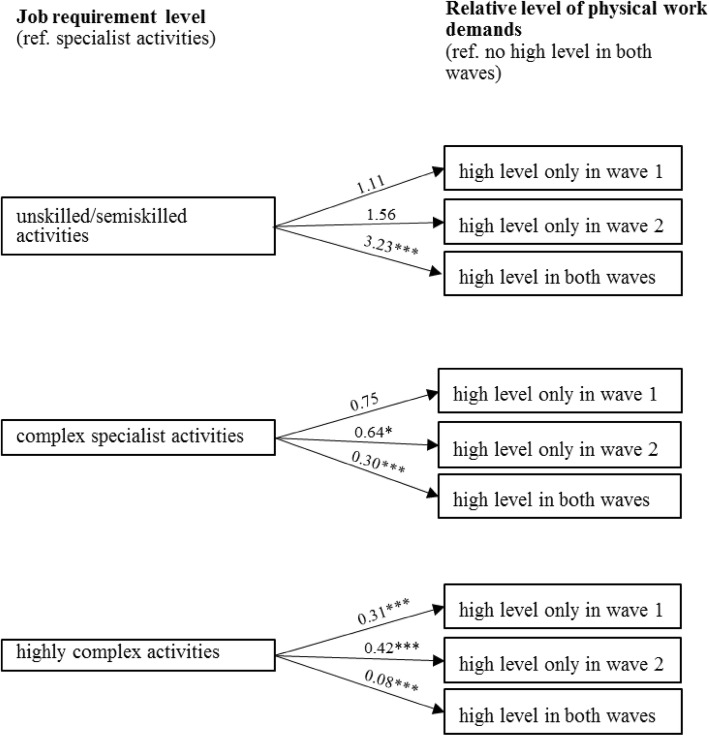
Supplement for Fig. 5: odds ratios. *n* = 3644; *p < 0.05, **p < 0.01, ***p < 0.001. The effects presented stem from logit models estimating the odds ratios of being in one of the categories compared to the reference category. Source: lidA waves 1 and 2, own calculations

## Data Availability

The lidA Survey Data (as a scientific use file) are available on request from the Research Data Centre of the German Federal Employment Agency: https://fdz.iab.de/en/FDZ_Individual_Data/lidA.aspx.

## Appendix

See Table 3 and Fig. 6.

**Table 3:** Multivariate analyses: direct and indirect effects on prospective physical health status (extended version of Table 1). Source: lidA waves 1 and 2, own calculations

	Dependent variable physical health score in wave 2		
	Whole sample	Men	Women
Direct effects			
Job requirement level^a^			
Unskilled or semiskilled activities	− 1.33*	− 1.95	− 1.02
Complex specialist activities	0.96**	1.14*	0.79
Highly complex activities	1.8***	1.79***	1.86***
Level of psychosocial work demands^b^			
High level only in wave 1	− 0.06	− 0.68	0.59
High level only in wave 2	− 1.82***	− 1.77*	− 1.76**
High level in both waves	− 1.24**	− 1.1*	− 1.26*
Level of physical work demands^b^			
High level only in wave 1	− 0.02	− 0.24	0.14
High level only in wave 2	− 0.97	− 1.25	− 0.75
High level in both waves	− 1.54***	− 1.23**	− 1.83***
High overcommitment	− 0.14	0.41	− 0.59
Year of birth: 1959	− 0.94***	− 1.14**	− 0.74*
Working full time	− 0.49	0.39	− 0.82*
Physical health score in wave 1	0.53***	0.54***	0.52***
Indirect effects			
Via level of psychosocial work demands^a^			
Unskilled or semiskilled activities	0.12*	0.03	0.11
Complex specialist activities	− 0.04	− 0.06	− 0.07
Highly complex activities	− 0.05	− 0.06	− 0.09
Via level of physical work demands^a^			
Unskilled or semiskilled activities	− 0.45***	− 0.16	− 0.68***
Complex specialist activities	0.33***	0.45**	0.16*
Highly complex activities	0.49***	0.59*	0.36**
Coefficient of determination (*R*^2^)	0.5	0.52	0.52
Standardized root mean squared residual (SRMSR)	0.04	0.04	0.04
*n*	3644	1666	1978

The analyses are based on multiple linear regression analyses. The table shows unstandardized regression coefficients

**p* < 0.05, ***p* < 0.01, ****p* < 0.001

^a^Reference category: specialist activities

^b^Reference category: no high level in both waves

## Footnotes

1 In our data, most employees’ vocational training matches their current job requirement level.

2 The choice of items is based on a factor analysis.

3 Accordingly, the workload rises the less often the employees sit at their workplace (Kroll 2011).
